# Gender and Racial Trends Among Vascular Neurology Fellowship Programs: By Design or By Default

**DOI:** 10.7759/cureus.17740

**Published:** 2021-09-05

**Authors:** Hamza Maqsood, Sadiq Naveed, Shifa Younus, Muhammad T Khan, Faisal Khosa

**Affiliations:** 1 Internal Medicine, Nishtar Medical University, Multan, PAK; 2 Psychiatry, Hartford Hospital - Institute of Living, CT, USA; 3 Neurology, Charleston Area Medical Center, Charleston, USA; 4 Radiology, Vancouver General Hospital, Vancouver, CAN

**Keywords:** gender disparity, healthcare, vascular neurology, racial disparity, under-represented minorities

## Abstract

Introduction

Benefits of increasing diversity in teams include the addition of different perspectives leading to increased innovation and creativity, faster problem solving, improved workforce morale, and reduced burnout leading to improved patient outcomes. This article reviewed the trend of gender and racial disparity in vascular neurology fellowship programs.

Methods

We retrospectively analyzed the data extracted from the Accreditation Council for Graduate Medical Education (ACGME)’s annual Data Resource Books from 2007 to 2019. ACGME cataloged gender as men and women and race/ethnicity was categorized as White/Non-Hispanic, Asian or Pacific Island, Hispanic, Black/Non-Hispanic, Native American/Alaskan, others, and unknown. Counts, proportions, relative, and absolute percentage changes were calculated to highlight trends in resident appointments over time and across the specialty of vascular neurology.

Results

The representation of females increased steadily; with a relative increase of 11.78% from the year 2007 to 2019. Race/ethnicity was reported starting from the year 2011. When averaged across the nine-year study period, 35% of the study sample was White (Non-Hispanic), followed by Asian/Pacific Islanders at 25%. The representation of Hispanics was 4.8%, Black/African Americans were 3%, Native Americans/ Alaskans were 0.23% and Others were 13% of the total study population. For 17.7% of the fellows, racial data were not known and was categorized as Unknown racial distribution.

Conclusion

Our study concludes that gender and racial disparity persists within the fellowship programs of vascular neurology. Effective strategies at individual, administrative, and national levels are needed to engage women and under-represented minorities in vascular neurology as a career choice.

## Introduction

Healthcare diversity is a fulcrum for raising healthcare standards, improving clinical outcomes, increasing patient satisfaction, and bringing innovation to society [1]. Healthcare needs are still catered by the homogenous care team, resulting in less-than-optimal care, clinical decision-making shortcomings, and poor financial outcomes in the pay-for-performance reimbursement model [2]. Despite changing social attitudes from assimilation models to valuing diversity, underrepresented minorities (URMs) face significant challenges. Existing literature has identified four underrepresented groups compared to their numbers in the general population: women, racial and ethnic minorities in medicine, sexual and gender minorities, and people with disabilities [3]. However, this problem is particularly concerning for the African American/Black and Hispanic/Latino minority groups, comprising 30.9% of the U.S. population compared to 9% of registered nurses and 8.5% of physicians from these racial minorities [4].

Diversity and inclusion of URMs raise optimism since women, African Americans, and Hispanics are more likely to serve the under-served populations [5-6]. The Liaison Committee of Medical Education (LCME) and Accreditation Council for Graduate Medical Education (ACGME) accreditation have focused on addressing gender and racial disparities in all facets. The Sullivan Commission report on diversity in the healthcare workforce has proposed three essential changes: change in the culture of health professions, new and non-traditional paths to healthcare, and commitment at leadership levels. These changes can become a catalyst for change and address current segregation and social exclusion [7].

Currently, the lack of diversity is evident across multiple subspecialties of medicine, NIH funding, professional societies, medical journal’s editorial boards, and clinical trials [8-12]. Existing literature reflects several factors and their complex interplay that maintain and support this gender and racial disparity. Isolation, lack of camaraderie among current fellows, discrimination, communication barriers, greater debt burden, work-life imbalance, lack of mentorship, lack of females and minorities in programs, challenges in making meaningful relationships, and difficulties associated with obtaining grants are some of the factors contributing to gender and racial disparities, [13-14].

Gutierrez and colleagues analyzed the profile of medical school enrollees, graduates, and neurology faculty, suggesting significant disparities for the Black/African American and Hispanic/Latinos [15]. Men were 1.4 times more likely to attain higher education and become tenured than female counterparts [15]. This gap was more pronounced for leadership roles since only 12% of chairs were women and were White/Caucasian [15]. These findings were corroborated by another study exploring gender and racial trends among neurology faculty [16]. In another study eliciting these trends among neurology residents, no appreciable change was observed for underrepresented minorities [8]. The trends among neurology faculty and residents paint an almost similar picture for gender distribution, raising concerns among current efforts to eliminate disparities and their effectiveness. It provides insight into disparities among the neurology workforce; there is limited evidence exploring these trends in vascular neurology.

We investigated the gender distribution of fellowship trainees in vascular neurology over the 13 years from 2007 to 2019 and the racial distribution over nine years from 2011 to 2019.

## Materials and methods

Data extraction

Two team members (HM, SY) independently extracted the data from the Accreditation Council for Graduate Medical Education (ACGME)'s annual Data Resource Books. In this paper, we extracted the data for the fellowship trainees in the discipline of vascular neurology. The data for gender distribution are available from 2007 to 2019, whereas the data for races/ethnicity are available from 2011 to 2019. The data were extracted into Microsoft Excel sheets (Microsoft Corporation, Redmond, WA). The third team member (SN) reviewed the extracted data for accuracy and discussed any discrepancies. Race/ethnicity was categorized as White/Non-Hispanic, Asian/Pacific Islander, Hispanic, Black (Non-Hispanic), Native American/Alaskan, Others, and Unknown. Gender was reported as men, women, and Not Reported.

Data analysis

We analyzed the data by gender and racial distributions and its temporal trends by year among vascular neurology fellows using Statistical Package for the Social Sciences (SPSS), version 27 (IBM Corp., Armonk, NY). Counts, proportions, and relative and absolute percentage changes were calculated to highlight trends in fellows appointments over time and across the discipline of vascular neurology.

Ethical approval

Ethical approval was not needed since there was no involvement of human or animal subjects. Our team sought all the information from the publicly available data of the ACGME.

## Results

**Figure 1:**
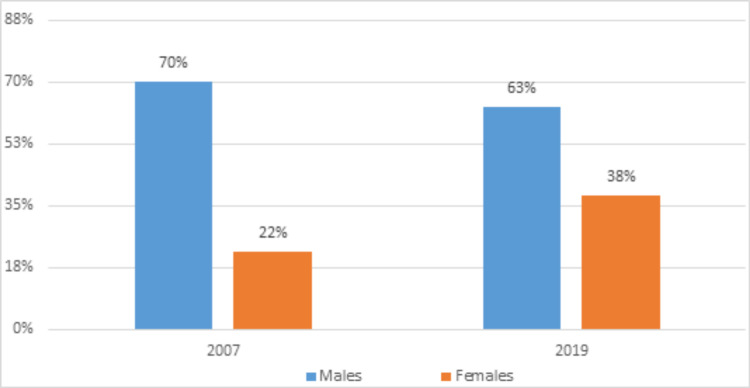
Gender differences at the beginning and end of our study period (i.e. 2007 to 2019)

When averaged over the 13-year study period, 66% of all vascular neurology fellows were male, whereas the representation of females was 30% (p<.001). Gender was not reported by 4% of the fellows. The representation of females increased steadily, with a relative increase of 15.18% from 2007 to 2019. In 2007, males accounted for 70% while females accounted for 22.22% of all fellows in vascular neurology, whereas, in 2019, males accounted for 64.5% while females accounted for 34.5% of all academic vascular neurologists (Figure 1).

For analysis of racial distribution, our study period ranged from 2011 to 2019. When averaged across the nine-year study period, 35% of the study sample was White (Non-Hispanic), followed by Asian/Pacific Islanders at 25%. The representation of Hispanics was 4.8%, Black/African Americans were 3%, Native Americans/Alaskans were 0.23%, and Others were 13% of the total study population. For 17.7% of the fellows, the racial data was unknown and categorized as Unknown racial distribution. The absolute change in racial distribution was highest for Asian/Pacific Islander (+20), followed by Whites (+18), Hispanics (+09), Black/African Americans (+2), Native Americans/Alaskans (00), and others (+3) (Table 1).

**Table 1:** Gender and racial differences as well as absolute and relative changes at the start and end of our study period

	2011 (%)	2019 (%)	Absolute Change (%)
White	36.3	36	-0.3
Asian/Pacific Islander	20.77	30.4	+9.63
Hispanic	00	7	07
Black/African Americans	6.5	5.4	-1.1
Native Americans/Alaskans	00	00	00
Others	14.28	11	-3.28
Unknown	20.77	11.7	+9
	2007 (%)	2019 (%)	Absolute Change (%)
Males	70	62.	-7.6
Females	22.22	37.	15.18

The yearly percentage of all neurosurgery residents by race and gender is shown in Table 2.

**Table 2:** Temporal trends for race and gender and absolute percentage change from the year 2007 to 2019

	2007 (%)	2008 (%)	2009 (%)	2010 (%)	2011 (%)	2012 (%)	2013 (%)	2014 (%)	2015 (%)	2016 (%)	2017 (%)	2018 (%)	2019 (%)
White					36.3	34	34.3	25.2	38.3	43.6	34	34.8	36
Asian/Pacific Islander					20.7	23.4	26.1	28.5	33	17.4	34	21.7	30.4
Hispanic					00	8.5	6.25	4.39	3.5	4.7	4.3	7	5.5
Black					6.5	1.06	2.08	1.1	1.78	4	1.7	5.4	5.4
Native American/Alaskan					00	00	00	1.1	00	0.8	00	00	00
Others					14.2	9.4	13.5	18.7	10	20.6	9.5	9.3	11
Unknown					20.7	22.3	17.7	21	13.4	8.73	16.5	21.7	11.7
Male	70	62	62.2	65	66.2	56.3	71	74	58	67	69	64	62.5
Female	22.2	26	28.7	27.6	23.7	42.5	27	26.3	42	33	30	34	37.5
Not Reported	8.3	12	09	7.4	10.4	1.06	2.1	00	00	00	01	02	00

## Discussion

We investigated the gender distribution of fellowship trainees in vascular neurology over the 13 years from 2007 to 2019 and the racial distribution over nine years from 2011 to 2019. Despite an increase in the total number of fellowship positions in vascular neurology, significant racial and gender differences persist.

When averaged across the 13 years of the study period, almost 66% of all neurosurgery residents were men; these findings are consistent with previous studies, both for vascular neurology and other surgical specialties [17-19]. A study published in 2017 reported that vascular neurology, having been accredited for 11 years, had grown 860%, averaging 78.18% more fellows each year. Despite an increase in fellowship positions, significant gender disparity exists, reported by only 26% of female residents in the study conducted by Williams et al. [20]. In our study, female trainees increased in proportion, and the absolute number of female trainees has also increased from 22.2% in 2007 to 34.5% in 2019. These findings are consistent with existing studies suggesting a suboptimal increase in the percentage of female trainees in vascular neurology [20]. However, it is worth noting that the percentage of women entering into other neurology sub-specialties is encouraging [20-21].

Factors affecting choosing a fellowship have been examined extensively in the literature on various medical and surgical specialties. McNutt. et al. described some important factors for both men and women: friendly training environment, communication level among fellows, variety, number of cases, and the quality of relationships with the mentor [22]. Similarly, Auriemma and Whitehair conducted a study on trainees of physical medicine and rehabilitation and determined the highest-ranked factors including the perceived happiness of current trainees, opportunities for hands-on training, and perceived camaraderie [23]. These studies found that the female applicants preferred programs with a higher percentage of female faculty.

Isolation, lack of camaraderie among current fellows, duration and rigors of fellowship, lack of early discipline related exposure, relationship with the mentor, chances of placement in a fellowship, limited research opportunities, work-life balance, pregnancy, parental leaves, implicit bias, harassment, and lack of same-gender mentor due to the lackluster representation of females at higher academic tiers are some factors that can sustain gender disparity [20]. The underrepresentation of women in vascular neurology is more a consequence of discrepancies in opportunities, mentoring, or conscious or unconscious bias on residents and faculty [20]. Pregnancy and parental leave are among the most important issues for female vascular neurology trainees and practicing vascular neurologists and contribute to a smaller number of female fellowship applicants and higher attrition rates of female trainees and vascular neurologists [24].

A comprehensive plan to advance and support women's careers in vascular neurology should be devised at all levels. At the mentorship level, it should be determined if there may be bias in how female trainees are perceived or treated if there are differences in the way the mentors assess the capability of female trainees. For research and leadership initiatives, female trainees should be provided with the opportunity to lead projects that align with their interests [21]. At the institutional level, the strength of female mentors and career scientists should be increased to engage women in leadership positions and provide examples of success along with the provision and support of workshops, leadership seminars, and programs that address gender bias in research careers and academic promotion. Nominate women for awards that target specific challenges faced by women. An equal opportunity for participation and recognition in the discipline should be given [21]. At the national level, support should be provided for research for understanding antecedents and sustainers of gender bias. Opportunities should be provided for women's speakership and to incentivize the inclusion of female researchers in large collaborative science grants [21]. Acknowledging and publicizing the promotion of female vascular neurologists (especially those in senior leadership positions) could inspire, encourage, and motivate women in their early careers to aspire and prepare for leadership positions in the future [25].

A similar trend is seen while comparing the representation of different races within the fellowship programs of vascular neurology in the US. When averaged across an eight-year study period, the White/Caucasian race was over-represented in many years, but Asians led the racial trend among all vascular neurology fellows. There can be several explanations for our findings. A previous study has attributed the increasing Asian faculty representation to a parallel increase in the total population of Asians in the US [26]. Over the past two decades, the Asian population has been the fastest-growing racial or ethnic group and is projected to become the second-fastest-growing group from 2014 to 2060 [20]. The Black/African American, Hispanic, and mixed-race physicians have even less representation in healthcare. Although the strength of Asian/Pacific Islander faculty is growing steadily, they still face challenges increasing their representation in leadership positions. Several barriers are faced by under-represented minorities (URMs) in getting training spots and promotions in faculty positions, including a greater debt burden, limited communication skills, as well as racial prejudice and discrimination at the workplace [20,25]. The recruitment and retention of ethnic minorities are also affected by the lack of minority preceptors in vascular neurology [25].

In any area of societal evaluation, the causes of racial differences are complex and multi-dimensional, with discrimination being one of them. Structural/ Institutional racism is as important as individual discrimination [27]. Structural racism reflects a system in which public policies, institutional practices, and other norms perpetuate racial group inequality. Most physicians are not explicitly racist and treat patients on an equal basis. Structural racism is insidious, and various studies document disparate outcomes for different races despite individual health care professionals [28].

There is also a need for intensive and systematic educational campaigns to highlight racial disparities in health care. The awareness levels of the public and professional community, especially the medical community, must be raised. Research should be done to identify strategies that are most effective to raise awareness and increase sensitivity to racial discrimination in neurology in general and vascular neurology in particular [27]. Another critical goal of medical education should be to increase the number of minority professionals. But current trends show that these goals are unlikely to be achieved. There has been very little increase in the proportion of vascular neurologists' URM backgrounds in the last years. Finally, to provide clinical care and conduct research that contributes to equity, we believe it’s crucial to “center at the margins.” Centering at the margins signifies that we should re-anchor our academic and health care delivery systems- specifically, diversifying the workforce, developing community-driven programs and research, and helping to ensure that URM gains positions of leadership [13].

The findings of this study indicate that further research is needed into the multifactorial reasons contributing to the decreased representation of URM in fellowship programs in vascular neurology.

Limitations

Our study has its share of limitations. It focused on data on fellows of vascular neurology and thus may have limited generalizability to other sub-specialties. Our study utilized a dataset that describes gender in a binary fashion. Finally, our study did not explore the combined effects of being a gender and a racial minority such as female Hispanic or Black residents.

## Conclusions

Our study concludes that gender and racial disparity persist within fellowship programs in vascular neurology. Efforts at all levels are needed to provide greater support for the females and for the careers of URM faculty to ensure their unbiased representation at all levels of academic neurology. Effective strategies at individual, institutional, and national levels can help increase the engagement of URM in choosing vascular neurology as their career.
